# Genome Sequences of Five Microviruses and a Murine Norovirus

**DOI:** 10.1128/MRA.00232-21

**Published:** 2021-05-27

**Authors:** Rui Zhou, Jun Yin, Juan Lu, Jianqiang Wang, Wen Zhang, Shixing Yang, Xiaochun Wang, Quan Shen

**Affiliations:** a School of Medicine, Jiangsu University, Zhenjiang, Jiangsu, People’s Republic of China; b Nanjing Customs District, Nanjing, Jiangsu, People’s Republic of China; c Intensive Care Unit, Jintan District Hospital of Traditional Chinese Medicine, Changzhou, Jiangsu, People’s Republic of China; KU Leuven

## Abstract

Murine norovirus is a fecal-orally transmitted pathogen in mice which belongs to the same genus as human norovirus. Microviruses are bacteriophages with small circular single-stranded DNA genomes, belonging to the family *Microviridae*. Here, we report the genome sequences of five microviruses and one murine norovirus obtained from the intestinal content of a lab mouse.

## ANNOUNCEMENT

Murine norovirus (MuNoV), a model used to study human norovirus, is a single-stranded positive-sense RNA (ssRNA) virus belonging to the *Caliciviridae* family (1). The *Microviridae* family, which is thought to be an important inhabitant of animal guts, can be divided into two subfamilies, *Bullavirinae* and *Gokushovirinae*, and at least two tentative subfamilies, including *Pichovirinae* and *Alpavirinae*, which have not been formally accepted by the International Committee on Taxonomy of Viruses (2–4).

The intestinal tissue of a laboratory mouse (BALB/c) with diarrhea from the Laboratory Center of the School of Medicine, Jiangsu University, was collected. The sample was resuspended in Dulbecco’s phosphate-buffered saline (d-PBS) and homogenized and filtered to remove eukaryotic and bacterial cell-sized particles (5). The filtrate was treated with a cocktail of DNases (Turbo DNase from Ambion, Baseline-ZERO from Epicentre, and Benzonase from Novagen) and RNase A (Fermentas) to digest unprotected nucleic acid at 37°C for 60 min, and then nucleic acid was extracted using the TaKaRa nucleic acid extraction kit. cDNA was synthesized using the SuperScript III first-strand synthesis system (Invitrogen) according to the instructions. The second strand of cDNA was synthesized using Klenow fragment DNA polymerase. Then, a library was constructed using the Nextera XT DNA sample preparation kit (Illumina) with dual barcoding for this pool. Sequencing was performed on the Illumina Miseq instrument using the MiSeq reagent kit version 2 (500 cycles) (6).

Raw data were processed according to the standard procedure, which included debarcoding, trimming, and assembling (7). In short, reads were debarcoded using vendor software from Illumina. Clonal reads were removed, and low-sequencing-quality tails were trimmed using a Phred quality score of 10 as the threshold. Adaptors were trimmed using the default parameters of VecScreen, which is NCBI BLASTn with specialized parameters designed for adaptor removal. The cleaned reads were *de novo* assembled with SOAPdenovo2 version r240 using a kmer size of 63 with default settings. Contigs and singlet reads were then compared against a customized viral proteome database using BLASTx with an E value cutoff of <10^–5^, where the virus proteome database was compiled using the NCBI virus reference proteome (ftp://ftp.ncbi.nih.gov/refseq/release/viral/), to which we added viral protein sequences from the NCBI nonredundant (nr) fasta file (only sequences taxonomically annotated as Virus kingdom). All tools were run with default parameters unless otherwise specified. Geneious software was used to check the circularity of the microviruses. The overlapping reads of the genomes at the start and end of the contigs confirmed their circular genomes. Five complete genomes of microviruses and one 6,131-bp-long partial genome of MuNoV with 5 gaps were generated. Seven sets of primers were used to close the gaps between sequences of MuNoV (Table 1). The Sanger sequences were assembled along with the initial contigs to bridge the gaps and obtain the complete genome using Geneious Prime software (version 2020.0.4). Putative open reading frames (ORFs) were predicted using Geneious Prime and NCBI ORFfinder. After multiple sequences were aligned with Clustal W, phylogenetic trees were generated with MrBayes version 3.2.7 with a mixed substitution model (8).

**TABLE 1:** Primers used to amplify the gaps of the MuNoV strain

Primer name	Sequence (5′-3′)	Polarity^*a*^	Location^*b*^	Length (bp)
F1	GTGAAATGAGGATGGCAAC	+	1–19	324
R1	AGATAGCCTTGTCAGACA	−	307–324
F2	CAAACTTGCCTCCACCAA	+	1118–1135	465
R2	CCAAGGGAGGCAGCAATT	−	1565–1582
F3	TGCACCTGTGTAGAAGAA	+	2232–2249	466
R3	CATCGTACTCCTCATCTGTG	−	2678–2697
F4	TGGGTTGTGATTGGGGTG	+	3438–3455	396
R4	GTTCTCAAGGCGGTTCTC	−	3816–3833
F5	ACCCTTGAACCGCAGAAG	+	3834–3851	341
R5	CAAGTCTCTTTCAGGGCATC	−	4155–4174
F6	GTGGCAACCTTCAAGATGAT	+	5965–5984	698
R6	CCTGTTGCCAAGCTTCCCA	−	6644–6662
F7	CCCCGCCTCTTTCAATTG	+	6619–6636	765
R7	AAAATGCATCTAATTACTAC	−	7364–7383

a +, forward primer; −, reverse primer.

b Location of the primer in the nucleotide residue of UJSMN01 (GenBank accession number MW018367).

**FIG 1:**
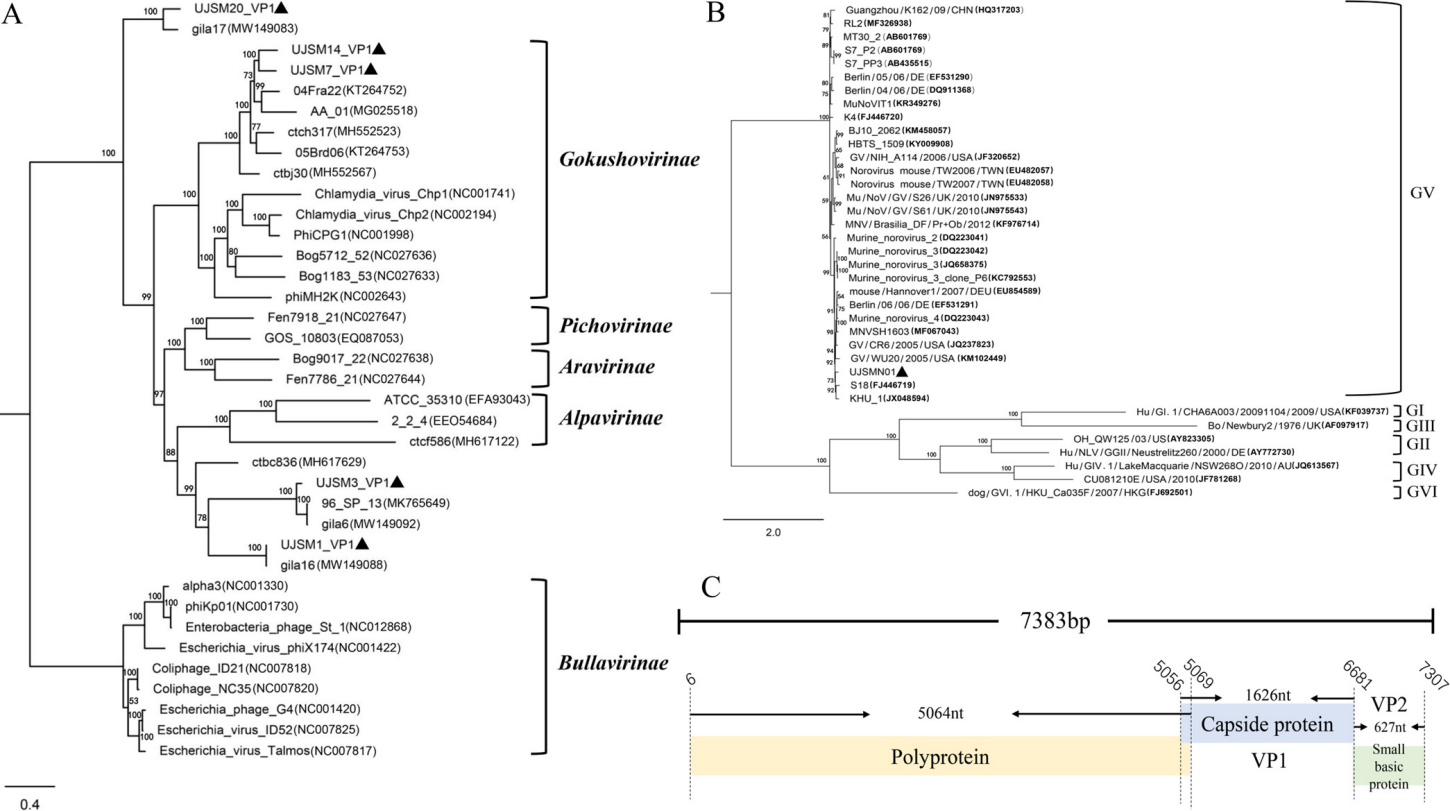
The phylogenetic diversity of microviruses and MuNoV and the genomic organization of MuNoV. (A) After aligning multiple sequences with Clustal W, a phylogenetic tree was created based on the amino acid sequence of major capsid protein VP1. The sequences in this analysis include the VP1 amino acid sequences of these five bacteriophage strains obtained in the current study and representative members of the previously identified subfamilies. The names of the subfamilies are shown beside the corresponding clades. (B) After aligning multiple sequences with Clustal W, a phylogenetic tree was created based on the major capsid protein of noroviruses, and the names of the genogroups are shown to the side. (C) Genomic organization of this MuNoV. The genomic positions of viral proteins are indicated.

The genomes of the 5 microviruses and 1 norovirus are 4,737 bp, 5,121 bp, 5,211 bp, 5,254 bp, 4,722 bp, and 7,383 bp long with G+C contents of 41.5%, 43.7%, 45.9%, 47.9%, 48.8%, and 56.8%, respectively. The vertical coverages of the genomes are 193×, 339×, 132×, 128×, 101×, and 34×, respectively. The phylogenetic tree based on the amino acid of major capsid protein VP1 indicated that these five strains belong to three different subfamilies; UJSM7 and UJSM14 belong to *Gokushovirinae*, and UJSM1/UJSM3 and UJSM20 belong to two potential new subfamilies, respectively (Fig. 1A).

The genome sequence of this MuNoV strain (named UJSMN01) is 7,383 bp long with three ORFs (Fig. 1C). Based on the phylogenetic tree, UJSMN01 belongs to genogroup V (GV) and shares the highest identity (91%) with a South Korean strain (GenBank accession number FJ446719) (Fig. 1B).

### Data availability.

These data are available in GenBank under BioProject accession number PRJNA666281, BioSample accession number SAMN16287582, and SRA accession number SRR12756396. The genome sequences of MuNoV and five bacteriophages have been deposited in NCBI GenBank under accession numbers MW018367, MW073821, MW073822, MW073823, MW073824, and MW073825, respectively.
